# Teachers’ Sense of Meaning Associations With Teacher Performance and Graduates’ Resilience: A Study of Schools Serving Students of Low Socio-Economic Status

**DOI:** 10.3389/fpsyg.2019.00823

**Published:** 2019-04-18

**Authors:** Shiri Lavy, Wesam Ayuob

**Affiliations:** Department of Leadership and Policy in Education, University of Haifa, Haifa, Israel

**Keywords:** teachers, graduates, sense of meaning at work, meaning, resilience, teacher-student relationships, low SES

## Abstract

Adolescents from lower socio-economic status (SES) often experience distress in their personal life as well as at school. Moreover, their ability to overcome such difficulties and pave their path to a higher SES depends, to a certain extent, on their ability to develop resilience despite their disadvantaged background. Acknowledging the critical contribution of teachers to students’ development, in the present study, we focused on teachers as agents who may influence graduates’ resilience, and on their sense of meaning at work–a resource these teachers may draw upon to increase their performance and contribute to their disadvantaged students and to their relationships with them. Specifically, we postulated that teachers’ sense of meaning at work will be associated with teachers’ performance and that teachers’ relationships with their students would mediate this association, as they serve as the main vehicle through which teachers impact their students. We further suggested that teachers’ sense of meaning would have long-term effects on students’ coping abilities, reflected in school graduates’ resilience levels. The study comprised 857 participants, teachers and graduates, from 30 Arab vocational schools in Israel, comprising mainly low SES students. Teachers (*N* = 436) completed self-report measures of their sense of meaning at work, relationships with students, and performance. Furthermore, to reveal potential long-term effects of teachers’ sense of meaning at work, school graduates (*N* = 421) completed measures of their relationships with teachers and resilience. Analyses indicated a significant association of teachers’ sense of meaning with their performance, which was mediated by teachers’ reports of their relationships with students. Furthermore, teachers’ sense of meaning at work and graduates’ perceptions of their relationships with the teachers were both significantly associated with graduates’ resilience. The findings highlight teachers’ sense of meaning at work as a potential contributor to their performance, which may also contribute to students’ resilience in lower SES schools. They point to teachers’ sense of meaning as a potential resource for teachers of lower SES students and highlight the importance of nurturing and developing it in various programs and practices (e.g., teacher training, teacher development, organizational routines).

## Introduction

Numerous scholars have underscored the centrality and importance of teachers’ beliefs and internal motivation (Beltman et al., 2011; Lavy and Bocker, 2018) and their striking effects on teachers’ performance and on their students’ self-perceptions and achievement (Rosenthal and Jacobson, 1968; Friedrich et al., 2015). Building upon these ideas, in the present research, we focus on a core motivator in teachers’ work that has scarcely been studied with quantitative tools–teachers’ sense of meaning at work. We explore associations between this motivator and teachers’ performance and relationships with students, and with graduates’ resilience. Teachers’ sense of meaning at work reflects their acknowledgment of the importance, significance, and purpose of their work (e.g., in terms of its potential positive impact on others; Martela and Pessi, 2018; Steger et al., 2012). It has been associated with different aspects of improved teacher functioning such as better teacher-student relationships, increased engagement, and decreased burnout (Lavy and Bocker, 2018; Lavy, under review). In the present study, we focused on the sense of meaning of a specific population of teachers, working in vocational schools that serve disadvantaged students from low socio-economic status (SES). These teachers face more challenges in their work, receive less pay, and are faced with increased student misbehavior and adjustment problems, as well as with the inherent difficulties of students from poor academic background (Hanushek, 2002; Duncan and Murnane, 2011, 2014). Thus, their sense of meaning may be an especially valuable resource to draw upon when facing daily work challenges, maintaining high-quality relationships with their students, and performing their job well. These teachers may also be especially valuable for these disadvantaged students, who have limited resources and whose education may be the main opportunity for social mobility, and a path out of poverty (Brown, 2013; Francis, 2015; Shoshana, 2019). In the present study, we focus on these teachers and explore the association of their sense of meaning with their performance. Furthermore, recent research indicates that teachers’ sense of meaning affects their relationships with students (Lavy and Bocker, 2018), and teachers’ relationships with their students are a key mechanism that predicts teacher functioning and resultant student achievement (Wubbels and Brekelmans, 2005; Roorda et al., 2011). We, therefore, suggest that teacher-student relationships are the primary mechanism underlying this association of teachers’ sense of meaning with their performance. Finally, to the best of our knowledge, the long-term effects of teachers’ sense of meaning have not been quantitatively examined to date. We therefore include, in the present study, an initial exploration of the association of teachers’ sense of meaning with school graduates’ resilience–a crucial capacity for young adults (especially in low SES). Including school graduates in the study in this way enabled an initial inquiry into potentially sustainable effects of teachers’ sense of meaning that persist after daily interactions with teachers are over. Exploring these correlates of teachers’ sense of meaning with teacher and student outcomes has notable implications for our understanding of teachers’ resources and their correlates and for developing inexpensive means of expanding these resources.

### Teachers’ Sense of Meaning at Work

A sense of meaning at work has been defined in various ways (Kaplan and Tausky, 1974; Schnell et al., 2013), typically focusing on aspects of work which provide individuals with a sense that their work is purposeful and significant (Steger et al., 2012; Martela and Steger, 2016; Martela and Pessi, 2018) and that they positively contribute to others (Grant, 2007, 2012; Littman-Ovadia and Steger, 2010). Research suggests that a sense of meaning can serve as a resource for increased motivation and improved functioning (Pessi, 2017). As noted by Kern et al. (2014), p. 501, people seem to “function best in both personal and work lives when they have a sense of meaning or purpose, defined in terms of having a direction, connecting to something larger than oneself, and feeling that what one does is valuable.” Empirical findings support these ideas, indicating associations of a sense of meaning at work with desirable personal outcomes such as well-being (Arnold et al., 2007) and fulfillment (Steger et al., 2012), and with desirable outcomes at work, such as increased career and organizational commitment, organizational citizenship behavior, job satisfaction, intrinsic work motivation, retention, and performance, and fewer manifestations of burnout, absence days and withdrawal behavior (Clausen and Borg, 2011; Pines, 2011; Steger et al., 2012; Littman-Ovadia et al., 2017).

A sense of meaning is considered a central motivation in teachers’ work, which is concerned with providing skills and knowledge and with assisting the development of others, for their benefit and for the benefit of the societies in which they live (Nias, 1989; Mayseless, 2016). Thus, it has been suggested that teachers’ sense of meaning can be an important resource for them in withstanding the daily hassles and organizational stressors (Pines, 2002; Korthagen, 2004, 2014; Guarino et al., 2006). Indeed, teachers’ sense of meaning has been associated with better health, increased life satisfaction (Kern et al., 2014), job satisfaction, and engagement, and decreased teacher burnout (Lavy, under review; Lavy and Bocker, 2018). Based on this literature, and on the perception of teachers’ sense of meaning as a core motivator (Oplatka, 2006; Osguthorpe and Sanger, 2013), in the present study, we suggest that teachers’ sense of meaning may have prominent effects on their work performance. This idea corresponds with previous research linking employees’ sense of meaning and acknowledgment of their perceived prosocial contribution to others with their performance (Grant, 2012; Littman-Ovadia et al., 2017).

*H1: Teachers’ sense of meaning at work will be positively associated with their reported performance*.

### Teachers’ Sense of Meaning and Teacher-Student Relationships

Teacher-student relationships are one of the most robust predictors of students’ well-being, motivation, social behavior, and academic engagement and achievement (Roorda et al., 2011; Gehlbach et al., 2012 meta-analysis; Wubbels and Brekelmans, 2005). Specifically, students’ feelings that their teachers care for them were associated with these students’ self-esteem, well-being, and school engagement (Lavy and Naama, under review), and teachers’ motivation to connect with their students predicted teachers’ effective teaching and effective social support of their students (Butler, 2012; Butler and Shibaz, 2014). These effects may be even more significant for relationships of teachers with students from low SES, as these students have fewer sources of support on which to lean in times of need and may have fewer available adult relationship figures (Johnson, 2008; Sanders et al., 2014). Furthermore, beyond their effects on specific students, teacher-student relationships can also contribute to teachers’ ability to perform well due to their potential contribution to class climate and to teachers’ internal resources (e.g., decreased burnout; Lavy, under review). Thus, they may decrease teachers’ work challenges and increase their ability to cope with challenges that do occur.

Teachers’ relationships with students are the main vehicle through which teachers’ prosocial contribution is achieved (Lavy, under review; Oplatka, 2006). Therefore, teachers who are more aware of their potential contribution to students and have a higher sense of meaning at work are expected to care-for and invest more in their students and thus have better relationships with them. Recent studies provide initial support for these ideas, linking teachers’ daily sense of meaning at work with the quality of daily teacher-student relationships (Lavy and Bocker, 2018) and showing association of teachers’ sense of meaning with their students’ perception of being cared-for (Lavy and Naama, under review). Thus, teacher-student relationships are expected to be associated with teachers’ sense of meaning and performance. Furthermore, since they reflect the main relational and motivational path for actualizing teachers’ sense of meaning and creating a positive impact on students (in turn one of the main meaningful goals of teachers’ work) (Lavy, under review; Oplatka, 2006), we expected teacher-student relationships to mediate the association between teachers’ sense of meaning association and their performance.

*H2: Teachers’ relationships with their students will mediate the association of teachers’ sense of meaning at work with their performance.*

### Teachers of Students From Low Socio-Economic Status

Teachers’ work may be of special importance for students from low SES, which typically introduces several challenges related to financial, psychological, social, and academic difficulties (Bradley and Corwyn, 2002; Sirin, 2005; Hanson and Chen, 2007). These difficulties commonly carry over from one generation to the next, as young adolescents rarely escape the vicious circle of poverty, due to their limited resources (Jones, 2003; Lott, 2012). Low SES adolescents are well aware of these issues, of their limited opportunities for social mobility toward a better future (Shoshana, 2016, 2017; Amir and Shoshana, 2018). Within this context, education is considered a possible route for social mobility and a path out of poverty–by providing knowledge and skills, and by facilitating internal capacities (e.g., resilience, self-efficacy, a sense of self-worth) required for attaining higher education, remunerative employment, and for enabling awareness and motivation to become participating partners in a fulfilling societal life (Almog and Perry Hazan, 2011; Brown, 2013; Stephens et al., 2014; Perry-Hazan, 2015). Teachers of students from low SES thus have an opportunity to notably impact their students’ life trajectory by helping them gain the knowledge, skills, and capacities required for better employment and fulfillment (Barber and Mourshed, 2007; Jennings and Greenberg, 2009).

Furthermore, teachers’ sense of meaning may be an especially valuable resource for teachers of low SES students, as their work environment is more challenging (Duncan and Murnane, 2011, 2014). Teachers in schools serving students from low SES confront more issues related to behavior difficulties, low social skills, and adjustment problems, and their retention and commitment tend to be notably lower (Hanushek, 2002; Duncan and Murnane, 2011), while teachers in high SES schools seem to suffer less from these issues (Stephens et al., 2014). Thus, in the present study, we focused on teachers of students in Arab vocational schools in Israel, serving students from low SES who are also an ethnic minority in Israel, a population which is considered underprivileged and with limited resources.

### Teachers’ Sense of Meaning and School Graduates’ Resilience

Because teachers’ sense of meaning is considered a core motivator in their work (Oplatka, 2006; Osguthorpe and Sanger, 2013) and is expected to impact teachers’ relationships with students and general performance, it is also expected to have positive effects on students–the primary beneficiaries of teachers’ work. Furthermore, the effects of teachers (and more generally–of schools) on students are expected to be maintained, at least to some extent, after graduation, thus providing graduates with the psychological capital and cognitive skills which will enable them to contribute to society and to overcome life’s challenges (Barton et al., 2013–McKinsey Report; National Research Council, 2013). This psychological capital is especially important for school graduates from low SES, who face both limited opportunities and steeper challenges. Thus, in the present research, we explored the potential association of teachers’ sense of meaning with school graduates’ capacities, among graduates from low SES. We focused on a specific capacity which is crucial for these graduates, namely resilience.

Resilience, defined as the “process and outcomes of coping in response to risk, adversity, or threats to well-being” (Johnson, 2008, p. 386), has received increasing academic and professional interest over the last few decades. This interest reflects a motivation to identify and advance processes which contribute to adaptive functioning, and to promote individuals’ thriving, even in the face of adversity and imperfect conditions, as experienced by some children, youth, and adults (Zolkoski and Bullock, 2012; Theron et al., 2015).

One of the factors shown to profoundly contribute to resilience is meaningful interpersonal relationships (Johnson, 2008; Rutter, 2013). Specifically, adolescents experiencing challenging life conditions were found to benefit from positive relationships with adult figures as in some cases these adolescents’ peer relationships can be harmful, and in some cases, such adolescents withdraw from antisocial peers in an attempt to reduce adversity (Sanders et al., 2014). School environments that foster caring and supportive relationships between students and school-based professionals were found to contribute to the resilience of adolescents who come from disadvantaged circumstances (Garmezy and Rutter, 1983; Zimmerman and Arunkumar, 1994; Stewart et al., 2004). The associations of supportive teacher-student relationships with student resilience were also evident in studies of other populations. For example, positive statements by teachers (Burnett, 1999), positive relations with teachers (Košir et al., 2007) and a supportive network of teachers and friends in school (Alva, 1991) were all related to resilience. Conversely, experiences of rejection in school predicted lower resilience among students (Narayanan, 2014). Thus, as teachers’ sense of meaning is expected to promote positive, supportive, teacher-student relationships, it is also expected to contribute to school graduates’ resilience.

*H3: Teachers’ sense of meaning will be positively associated with school graduates’ resilience.*

### Summary of the Research Hypotheses

**Figure 1:**
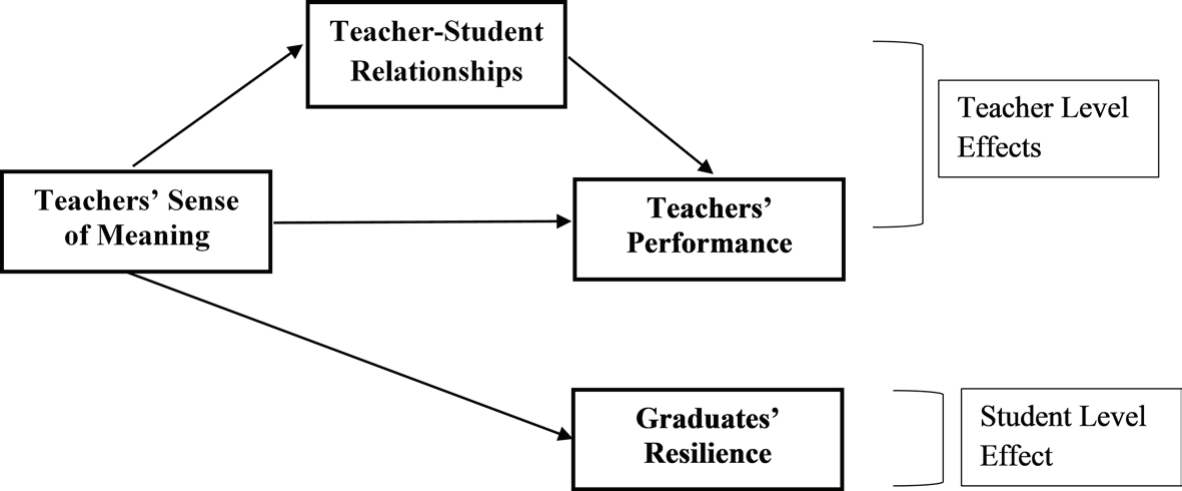
The theoretical model.

In sum, in the present study, we focused on teachers of students from low SES who attend vocational schools in Israel and examined the association of their sense of meaning with their performance (H1) and the role of their relationships with their students in mediating this association (H2). We also sought to provide an initial exploration of the long-term effects of the sense of meaning of these teachers on students, while exploring its correlation with school graduates’ resilience (H3). The integrative theoretical model is presented in Figure 1.

## Materials and Methods

The research hypotheses were examined in a cross-sectional study of schoolteachers and young school graduates randomly selected from 30 vocational schools in Israel, serving students from low SES. This design enabled examining the hypotheses related to teachers’ reports (i.e., the associations of teachers’ sense of meaning with their performance and relationships with students). It also enabled an initial exploration of the connection of teachers’ sense of meaning with an important personal capacity of the school graduates (despite its cross-sectional and reflective nature).

### Participants

The study comprised 857 participants: 436 teachers and 421 school graduates, from 30 Arab vocational schools in Israel (10–15 teachers and 10–15 graduates from each school, randomly sampled from the relevant lists). The teachers’ sample comprised more men than women (67.9%, typical to vocational schools in Israel). Teachers’ average age was 39.66 (*SD* = 10.24), their average tenure was 15.73 years (*SD* = 8.56 years), and they taught an average of 19.82 hours per week (*SD* = 8.7). Most teachers completed higher-education professional programs (57%) and/or had a master’s degree (36%). The others had a bachelor’s degree or a high-school graduation diploma (~1% each). The school graduates who participated in this study completed their studies in the 1–3 years prior to the study; their ages ranged from 18 to 20 (*M* = 18.63, *SD* = 0.53); and most of them were men (69.1%).

### Procedure

After receiving approval from the Institutional Review Board, we contacted school principals of Arab vocational schools in Israel from a list of relevant schools and asked their approval to contact teachers and graduates from their schools. Most principals (>85%) approved, allowing a research assistant to invite potential participants to voluntarily participate in the study (with no monetary compensation). Those who agreed (above 85% of the teachers and above 80% of the graduates) received a longer explanation about the study, provided their consent, and answered a few demographic questions and a brief survey including the measures described below. They were then thanked for their participation and debriefed.

### Teachers’ Measures

All the teachers’ questionnaires were administered in Hebrew, due to the high proficiency of the schoolteachers in Hebrew, and the complexity of achieving accurate (reliable and valid) written Arabic translation and ensuring that it is understood by all teachers (written Arabic is different from the spoken Arabic dialect in Israel).

*Work as Meaning Inventory* (WAMI; Steger et al., 2012) Hebrew version (Littman-Ovadia and Steger, 2010) was used to assess teachers’ sense of meaning at work. This 10-item measure taps participants’ positive meaning at work (e.g., *I found a significant career*), greater-good motivations (e.g., *I know my work generates a positive change in the world*), and meaning-making through work (e.g., *My work helps me better understand myself*). Participants rated the extent to which each item is true for them on a five-point Likert-type scale ranging from *1 (absolutely untrue)* to *5 (absolutely true)*. The scale showed satisfactory internal reliability (Cronbach’s *α* = 0.80), although slightly lower than in other studies in Israel (e.g., Cronbach’s *α’s* = 0.91–0.95 in Lavy and Naama, under review; Littman-Ovadia et al., 2017).

*Teacher-Student Relationships Questionnaire* (TSR; Gehlbach et al., 2012; Hebrew: Lavy and Bocker, 2018) was used to assess teachers’ perception of the quality of their relationships with their students. This questionnaire was originally developed to measure teachers’ relationship with a specific student, but it has also been used as a measure of teachers’ general perceptions of their relationships with students (Lavy and Bocker, 2018; Lavy and Naama, under review). It was used in this manner in the present study as well. The questionnaire comprises nine positive items (e.g., *How respectful are the students towards you*?) and five negative items (e.g., *How often do the students ignore something you say*?). Each item was ranked on a scale ranging from 1 (*not at all/never)* to 7 (*very much/all the time).* The internal reliability of the scale in the present study was satisfactory (Cronbach’s *α* = 0.84) and similar to that achieved in previous studies (for example, Cronbach’s *α*’s = 0.72–0.95; Gehlbach et al., 2012).

*Teacher performance* was assessed with an integrated measure (Goodman and Svyantek, 1999), comprising behaviors related to in-role performance, extra-role contributing behavior (based on organizational citizenship behavior measure), and withdrawal behaviors. The measure comprises 25 items, related to three main factors: Altruism (e.g., *Help other employees with their work when they have been absent*), conscientiousness (e.g., *Exhibit punctuality arriving at work on time in the morning and after lunch breaks*), and in-role performance (e.g., *Fulfill all the requirements of the job*). Teachers rated the frequency in which they behave in the way described in each sentence, on a response scale ranging from *1 (very rarely)* to *4 (very often).* The scale’s internal reliability (across the three factors) was good (Cronbach’s *α* = 0.86), and in line with previous studies (e.g., Cronbach’s *α* = 0.86 in Lavy and Littman-Ovadia, 2017).

### School Graduates’ Measures

The school graduates’ questionnaires were administered verbally in Arabic, to avoid misunderstandings related to language (the students were not proficient in Hebrew and written Arabic, which is different from the spoken Arabic dialect in Israel).

*The Brief Resilience Scale* (Smith et al., 2008) was used to assess graduates’ resilience. The six scale items tap individuals’ ability to recover after negative events (e.g., *I tend to bounce back quickly after hard times*). Graduates rated their agreement with each item on a scale ranging from *1 (strongly disagree)* to *5 (strongly agree).* After omitting one item that was frequently misunderstood (item 3: *It does not take me long to recover from a stressful event*), the internal reliability of the remaining five items was acceptable (Cronbach’s *α* = 0.70), although slightly lower than reported in the scale development (Cronbach’s *α*’s = 0.80–0.91 in the scale development).

The *Teacher–Student Relationships Questionnaire* (TSR; Gehlbach et al., 2012; Hebrew: Lavy and Bocker, 2018) described above was also used to assess graduates’ perceptions of their relationships with their teachers. Here, the students’ version was used, and graduates rated the extent to which their schoolteachers generally fit the items’ descriptions (e.g., *How friendly were the teachers towards you?*). The measure’s internal reliability was good (Cronbach’s *α* = 0.87).

### Data Analysis

After initial examination of the variables’ means, and standard deviations, we explored zero-order correlations among the study variables, while also examining the associations of teachers’ sense of meaning with their performance (H1). Then, in order to further explore the hypothesized mediation model (H2), in which teacher-student relationships mediate the association of teachers’ sense of meaning at work with their performance, we used PROCESS code for SPSS (Model 4; Hayes, 2012, 2013). This analysis enables using bias-corrected bootstrapping methods, which make no assumptions of normality (MacKinnon et al., 2004), and are considered most suitable for exploring mediation in cross-sectional studies such as the present one (Fritz and MacKinnon, 2007; MacKinnon et al., 2007). In the presented analysis, teachers’ sense of meaning at work was entered as the independent variable, teachers’ performance was the dependent variable, and teachers’ reports of the quality of their relationships with their students were the mediating variable. Teachers’ workload (i.e., number of teaching hours per week), tenure, and number of years of education were entered as control variables.

Next, examination of the hypothesis linking teachers’ sense of meaning with school graduates’ resilience (H3) required associating teachers’ data with graduates’ data. This kind of multi-level analysis can be conducted while using hierarchical linear modeling (HLM). Initial interclass correlation analysis of graduates’ resilience ratings supported the need for exploring multi-level effects–including individual-level effects as well as school-level effects (*ICC* = 0.58; *p* < 0.01). To enable this kind of HLM analysis, while assuming that each school graduate has studied with several teachers from their school and each teacher had relationships with several school graduates, we first aggregated teachers’ data at the school level. This enabled linking teachers’ data with graduates’ data: graduates’ data were entered to the model at level 1 (the individuals’ level), and the aggregated teachers’ data were entered at level 2 (the school level). Subsequently, graduates’ resilience was entered to the equation as the dependent variable, teachers’ sense of meaning at work was entered as a level-2 independent variable, and students’ perceptions of their relationships with teachers were entered as level-1 control variable. Finally, students’ age and gender were also entered as control variables.

## Results

The variables’ means, standard deviations, and correlations are presented in Table 1. The zero-order correlations provide initial support for the first two research hypotheses, while indicating significant associations of teachers’ sense of meaning at work with their performance (H1; *r* = 0.50, *p* < 0.001) and with their relationships with students (H2; *r* = 0.61, *p* < 0.001). The correlations among school graduates’ variables indicate a significant association of the self-reported quality of their relationships with schoolteachers, with their resilience levels (*r* = 0.38, *p* < 0.001).

**Table 1:** Means, standard deviations, and correlations among teacher variables and graduate variables.

	Mean	*SD*	1	2	3
**Teachers’ data**					
1. Sense of meaning at work	4.23	0.55			
2. Teacher-student relationships (teachers’ reports)	5.44	0.70	0.61***		
3. Performance	3.41	0.39	0.50***	0.43***	
**Graduates’ data**					
1. Resilience	3.10	0.54			
2. Teacher-student relationships (graduates’ reports)	5.43	0.98	0.38***		

*** *p* < 0.001.

**Figure 2:**
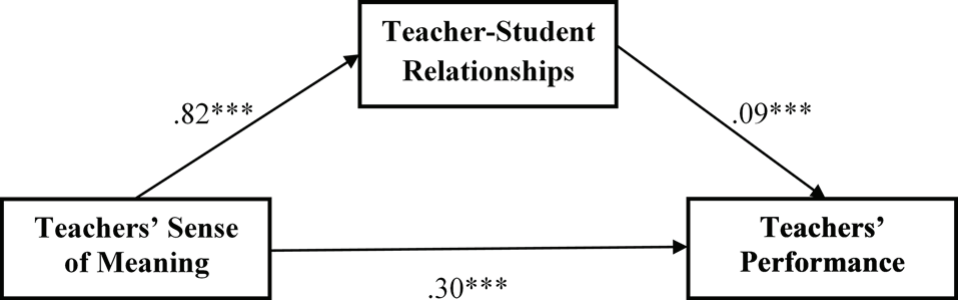
The mediation model.

In line with these zero-order correlations, the mediation analysis results showed that even when controlling for teachers’ workload, tenure, and education, teachers’ sense of meaning at work was still significantly associated with teachers’ performance (*B* = 0.30, *p* < 0.001) and was even more strongly associated with teacher-student relationships (*B* = 0.82, *p* < 0.001). The mediation analysis (Figure 2) supported the research hypothesis (H2) and indicated significant direct paths from teachers’ sense of meaning at work to their performance, as well as a significant indirect path *via* teacher-student relationships [*B* = 0.08; 95% *CI* (0.04, 0.12), note that zero was not within the 95% confidence interval, supporting the mediation hypothesis]. HLM examination of H3 indicated that teachers’ sense of meaning at work and graduates’ perceptions of the quality of their relationships with their teachers were both significantly associated with graduates’ resilience (Table 2). These effects were maintained when omitting one of the independent variables and when controlling for the demographic variables.

**Table 2:** Unstandardized HLM coefficients predicting graduates’ resilience from teachers’ sense of meaning and teacher-student relationships (student reports).

	Coefficient	Standard error	*T*-value
**Student level (level 1)**			
Age	0.09	0.06	1.66
Gender	0.02	0.09	0.18
Teacher-student relationships (graduates’ reports)	0.24	0.03	7.56***
**Teachers/school level (level 2)**			
Teachers’ sense of meaning	1.19	0.49	2.41*

* *p* < 0.05;

*** *p* < 0.001.

## Discussion

The present study focused on the sense of meaning of teachers working with students from low SES. Its findings revealed significant associations of teachers’ sense of meaning with their performance, pointed to teachers’ relationships with students as a mechanism underlying this effect-and further linked these teachers’ sense of meaning with school graduates’ resilience. These findings highlight the potential importance of teachers’ sense of meaning for teachers serving disadvantaged students and have implications for theory and practice, as well as for future research. Although a sense of meaning at work has consistently been suggested to be an important work resource (Kahn, 1990; Crawford et al., 2010) and was argued to be crucial for teachers’ well-being and functioning (Pines, 2002), its correlates and effects have only rarely been explored empirically (Crawford et al., 2010; Bakker and Demerouti, 2014). The present study provides quantitative evidence for its association with teachers’ and school graduates’ variables and sheds light on a mechanism underlying its potential effects. Furthermore, the study is among the very few quantitative studies exploring the sense of meaning of teachers serving students from low SES and the first conducted among vocational schools’ teachers.

The findings linking teachers’ sense of meaning with their performance correspond with other studies indicating associations of employees’ sense of meaning with desirable feelings and attitudes at work, such as increased job satisfaction (Littman-Ovadia and Steger, 2010), happiness (Chan, 2009), and other indicators of employees’ well-being (Clausen and Borg, 2011). They further establish the link between a sense of meaning at work and performance–an important behavioral aspect, which is closely related to organizational productivity. In a similar vein, the present study’s findings linking teachers’ sense of meaning at work with their self-reported relationships with students fit well with previous findings indicating daily impact of teachers’ sense of meaning on their daily relationships with students (Lavy and Bocker, 2018) and suggest that these effects may be broader, extending beyond the daily level.

The mediation model, indicating that teachers’ perceptions of their relationships with their students mediate the association of teachers’ sense of meaning with their performance, sheds light on the psychological and interpersonal processes underlying potential effects of teachers’ sense of meaning. It supports the theoretical idea that teacher-student relationships are a primary path for actualizing teachers’ sense of meaning, based on the notion that teacher-student relationships are one of the main vehicles through which they can positively affect their students (Lavy and Bocker, 2018). This theoretical understanding suggests teachers’ sense of meaning as a potential antecedent that may contribute to better teacher-student relationships, which are crucial for student well-being, development, and achievement (Hattie, 2008; Jennings and Greenberg, 2009) and that teacher-student relationships, in turn, may feed teachers’ need to feel the positive impact of their work and contribute to their performance.

The association of teachers’ sense of meaning with school graduates’ resilience (even when controlling for demographic variables and for teacher-student relationships effects) provides a first, initial indication of possible long-term effects of teachers’ sense of meaning on their students after graduation. This finding corresponds with previous findings pointing to associations of teachers’ sense of meaning with students’ self-esteem, well-being, and school engagement (Lavy and Naama, under review) and suggests the possible impact that teachers’ sense of meaning may have on students. Furthermore, the association of teachers’ sense of meaning with school-graduates’ resilience was not examined at the individual teacher level but rather at the teachers’ group level, suggesting a possible effect of school culture supporting creation and maintenance of a high sense of meaning among teachers. This interesting finding emphasizes the need for further examination of the long-term effects of teachers’ sense of meaning.

### Practical Implications

The study’s findings may have practical implications for teachers, principals, school counselors, and policy makers, in developing and adopting strategies that may contribute to teachers’ functioning and to their students’ development. The findings point to the importance of teachers’ sense of meaning at work and suggest the need to acknowledge it in teacher recruitment and development processes. This need can be met by creating organizational mechanisms and programs that foster teachers’ sense of meaning and nurture its development in teacher training, teacher development programs, and daily practices. These implications may be especially relevant to schools serving students from low SES, whose teachers confront especially challenging difficulties related to student misbehavior and low social skills, and whose teachers retention and commitment tend to be low (Hanushek, 2002; Duncan and Murnane, 2011). Although these teachers face more challenges, they also have fewer resources available to cope with these challenges. Thus, revealing a potential psychological resource that can contribute to their functioning is valuable.

Furthermore, most interventions for underprivileged students focus on providing additional resources to these students, such as extra mentoring and tutoring (Gándara, 2001). Although some of these interventions were proven effective, they were typically costly and had questionable sustainability and thus were only available to a small percent of underprivileged students (Gándara, 2001). The present study suggests an additional possible path for interventions, focusing on psychological resources of teachers of underprivileged students. It specifically points to teachers’ sense of meaning at work as a potential enhancer of teachers’ ability to connect with their students, to perform well, and to contribute to their students’ capacity to cope with challenges they encounter after graduation. Relevant interventions can include, for example, simply asking teachers to reflect on meaningful events that happened to them during the day/week (based on Lavy, under review), encouraging teachers to structure their work in ways that would make it more meaningful (for details, see Wrzesniewski and Dutton, 2001; Demerouti, 2014), and incorporating teacher training programs that focus on bringing teachers back in touch with their sense of mission and exploring ways to pursue it (see details in Korthagen, 2004; Korthagen and Vasalos, 2005).

### Limitations

The study’s implications should be considered while acknowledging its limitations. As a cross-sectional study attempting to link concurrent reports of teachers and school graduates, it is based on school graduates’ memories (which can be inaccurate) and link them with current teacher reports about their experiences and feelings (which may change over time). Furthermore, because each teacher has taught several students, and each school graduate has studied with several teachers, teachers’ data were aggregated to the school level, not considering individual teacher-student relationships (which may be impactful). The fact that the association was significant despite these limitations suggests that the actual link between specific teachers and specific students (who later graduate) may actually be stronger. However, a sound, longitudinal studies of teachers and students (who later become school graduates) is required in order to validate these effects and also indicate the causal effect of teachers’ sense of meaning on teacher performance and graduates resilience (showing that it is not a mere correlation driven by another variable, for example). Such studies would optimally include, in addition to self-report measures, peer-ratings, and behavioral measures of teacher functioning and student outcomes. Another caveat is that the study was conducted in a specific population of teachers and students in Arab vocational schools, comprising mostly students from a minority group from low SES. These characteristics may amplify (or otherwise affect) the effects of teachers’ sense of meaning. The correlates of teachers’ sense of meaning may be different in schools serving other populations (in terms of their SES, cultural background etc.), and additional research is required in order to evaluate the generalizability of the present study’s findings to these contexts.

Despite its limitations, the study provides initial evidence for associations of teacher sense of meaning with desirable outcomes for teachers and students and suggests that it may serve as an important resource for teachers, that its nurturance can improve teaching and eventually contribute to the development of students’ personal skills and competencies (which can also contribute to their academic achievement). As such, the study highlights the need for further exploration of potential effects and antecedents of teachers’ sense of meaning, especially in challenging work circumstances. It is our hope that the compelling findings of the present study will encourage researchers to further study teachers’ sense of meaning and its effects, as an understudied potential resource.

## Ethics Statement

This study was carried out in accordance with the recommendations of the University of Haifa Ethics Committee, with written informed consent from all teacher participants, in accordance with the Declaration of Helsinki. For graduate participants who had difficulty with the written consent form–the form was read aloud, and they provided oral consent (which was recorded in writing). The protocol was approved by the University of Haifa, Faculty of Education Ethics Committee (approval number: 130/17).

## Author’s Note

This manuscript is based on the Master’s Thesis of Wesam Ayuob, initiated and supervised by Shiri Lavy.

## Author Contributions

SL and WA contributed conception and design of the study. WA was responsible for the data collection, and organizing the database. WA and SL performed the statistical analyses and wrote an initial outline of the manuscript. SL wrote the manuscript.

### Conflict of Interest Statement

The authors declare that the research was conducted in the absence of any commercial or financial relationships that could be construed as a potential conflict of interest.
